# Case report: Envafolimab causes local skin necrosis

**DOI:** 10.3389/fimmu.2024.1336311

**Published:** 2024-03-22

**Authors:** Jing Jing Liu, Xiao Ya Xu, Huan Han, Tong Wang, Wei Zhang, Jing Cui, Maksim Semenov

**Affiliations:** ^1^Yu Lin City First Hospital (Yan An University Second Affiliated Hospital) Pharmaceutical Department, Yu Lin, Shaanxi, China; ^2^Shaanxi Province People’s Hospital, Pharmaceutical Department, Xi’An, Shaanxi, China; ^3^Yu Lin City First Hospital (Yan An University Second Affiliated Hospital) Department of Oncology, Yu Lin, Shaanxi, China; ^4^Medical Affairs Department, Proswell Medical International Contract Research Organization (CRO), Beijing, China

**Keywords:** PD-L1, envafolimab, humanized monoclonal antibody, subcutaneous, skin necrosis, adverse drug reaction, case report

## Abstract

Envafolimab is a Chinese domestic innovative fusion of a humanized single-domain programmed death-ligand 1 (PD-L1) antibody (dAb) and human immunoglobulin IgG1 crystalline fragment (Fc) developed for subcutaneous injections. It was granted conditional market authorization by the China National Medical Product Administration (NMPA) in December 2021. Envafolimab is used to treat adult patients with previously treated microsatellite instability—high (MSI-H) or deficient mismatch repair (dMMR) advanced solid tumors, including patients with advanced colorectal cancer disease progression who were previously administered fluorouracil, oxaliplatin, and irinotecan, as well as other patients with advanced solid tumors who experienced disease progression after receiving standard treatment and had no other alternative treatment options. However, the lack of post-marketing clinical trial data requires conducting more clinical studies on the safety and efficacy of envafolimab in order to provide scientific basis and a reference for future therapeutic applications. In this paper, we report a case of severe skin necrosis and bleeding in the area of injection after subcutaneous administration of envafolimab in a patient diagnosed with hepatocellular carcinoma. We discuss issues that must be considered before administration of a PD-L1 inhibitor subcutaneously, which could induce immune mechanisms leading to skin necrosis in the area of injection.

## Introduction

Envafolimab is a Chinese domestic innovative fusion of a humanized single-domain programmed death-ligand 1 (PD-L1) antibody (dAb) and human immunoglobulin IgG1 crystalline fragment (Fc) developed for subcutaneous injections. It was granted conditional market authorization by the China National Medical Product Administration (NMPA) in December 2021 (1).

Envafolimab is used to treat adult patients with previously treated microsatellite instability—high (MSI-H) or deficient mismatch repair (dMMR) advanced solid tumors, including patients with advanced colorectal cancer disease progression who were previously administered fluorouracil, oxaliplatin, and irinotecan, as well as other patients with advanced solid tumors who experienced disease progression after receiving standard treatment and had no other alternative treatment options.

The molecular weight of envafolimab single-domain antibody is 80 kDa, which is half the molecular weight of most regular antibodies. It demonstrates quicker penetration and more effective saturation of the tumor tissue, as well as high stability and water solubility (2, 3). With these advantages, envafolimab became the first PD-L1 antibody for subcutaneous delivery. This route of administration improves convenience of use and also allows the use of this medication in patients with venous collapse or peripheral venous stenosis (4). It also shortens the administration time and helps avoid infusion reactions, which are widely encountered during intravenous injections. According to its instruction for use, the incidence of injection site reactions does not exceed 5% after infusion, which include rash, pain, swelling, dermatitis, discoloration, and erythema.

However, the lack of post-marketing clinical trial data requires conducting more clinical studies on the safety and efficacy of envafolimab in order to provide scientific basis and a reference for future therapeutic applications. Herein, we present a case of local skin necrosis and subcutaneous bleeding at the site of injection after subcutaneous administration of envafolimab in a 52-year-old patient diagnosed with hepatocellular carcinoma. Our case report also supports the need for further investigation into the potential use of subcutaneous infusions of immune checkpoint inhibitors (ICPIs) and the potential side effects related to this administration route.

## Case presentation

**Figure 1:**
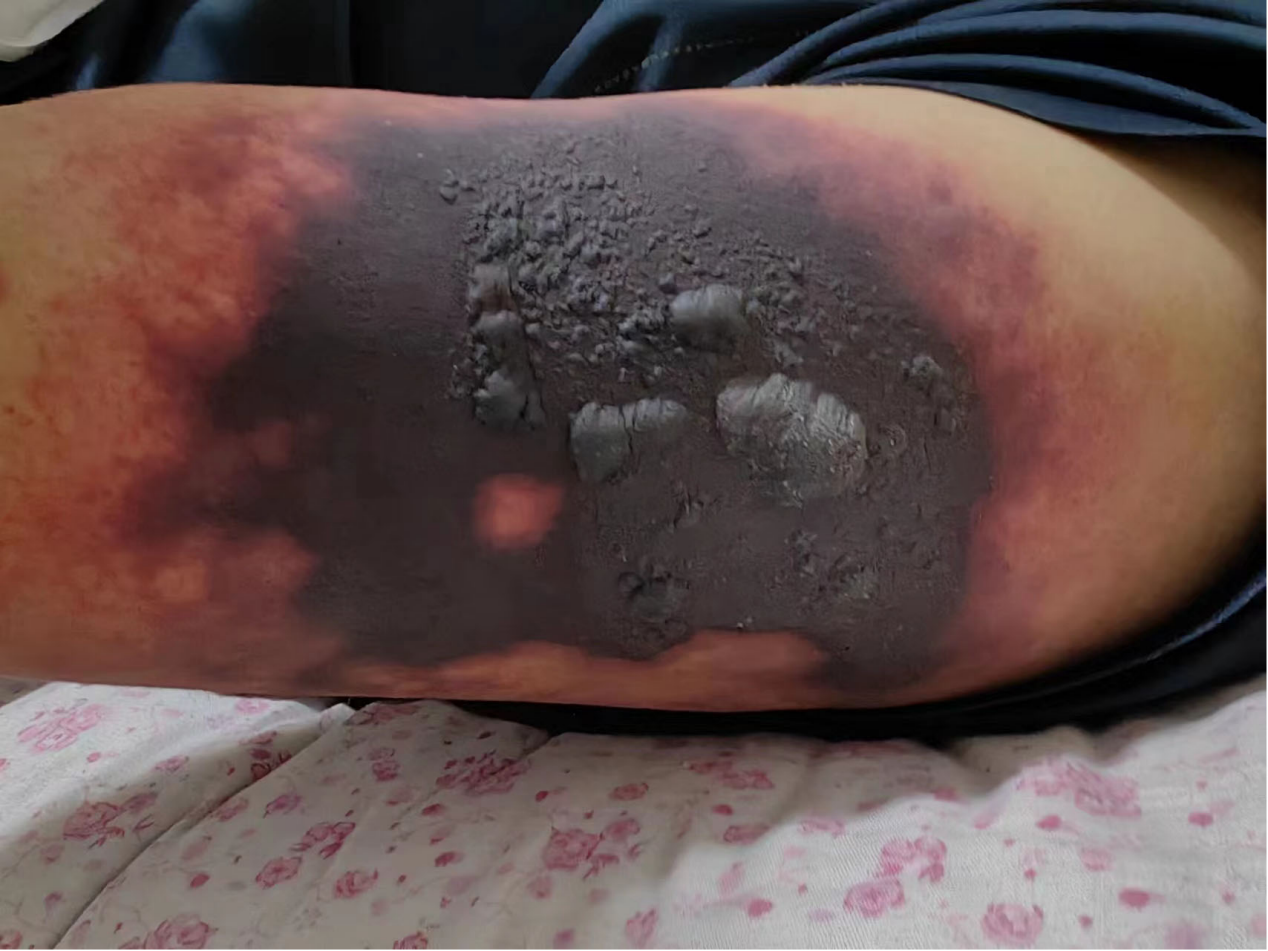
Image of the skin necrosis area in the left arm.

**Figure 2:**
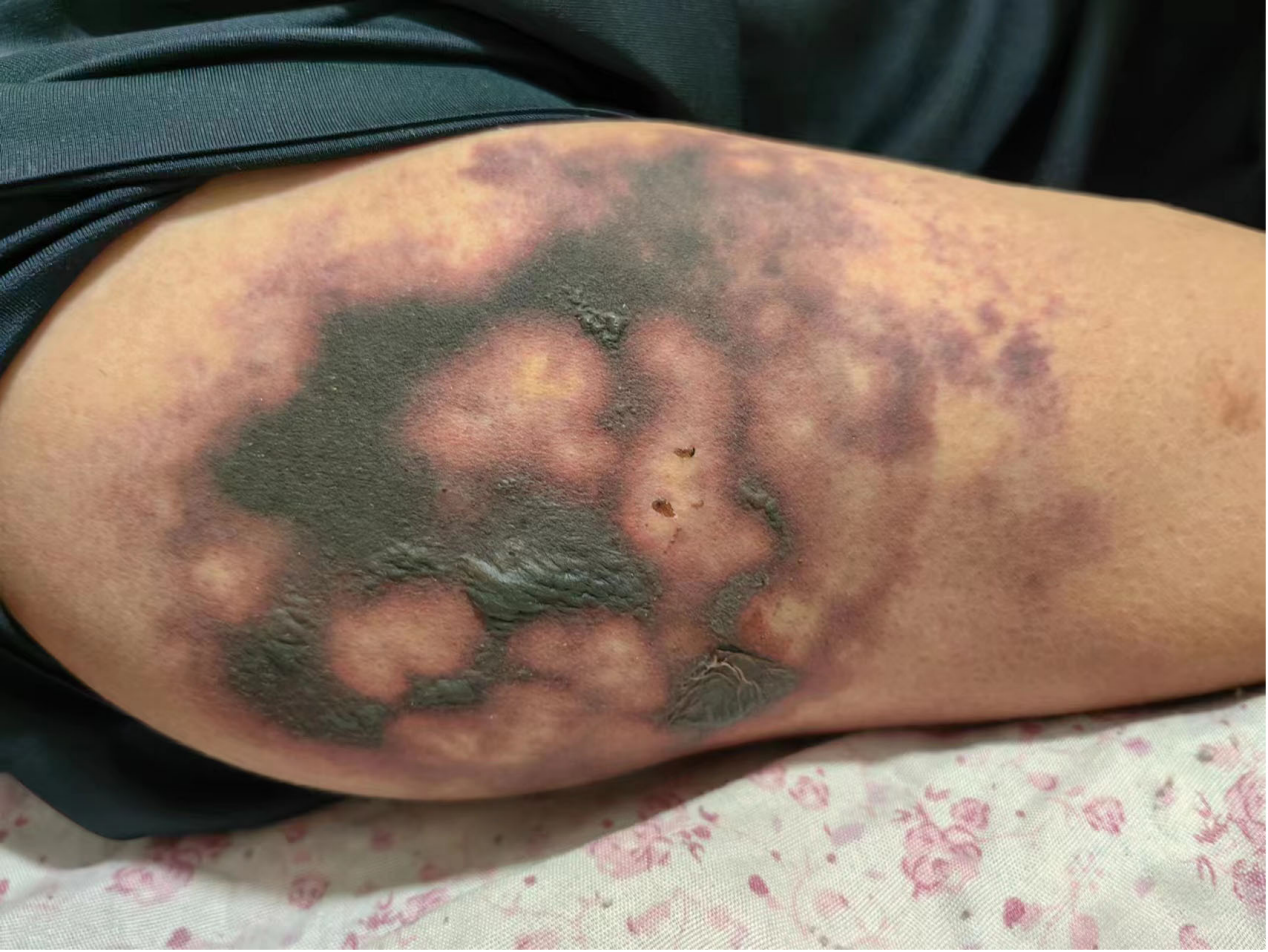
Image of the skin necrosis area in the right arm.

In May 2023, a 52-year-old male patient (height, 174 cm; weight, 58 kg) was brought to the emergency department. His platelet count was 47 × 10^9^/L (range, 100–300 × 10^9^/L), hepatitis B surface antigen was 250.79 cutoff index (COI; range, <0.03 COI), hepatitis B e antigen was 2.70 COI (range, <1 COI), hepatitis B e antibody was 51.50 Inh% (range, <50 Inh%), and hepatitis B core antibody was 462.90 COI (range, <1 COI). The patient had a past medical history of hepatitis B for about 15 years and antiviral and hepatoprotection therapy. The specific drugs were unknown. The patient reported no history of food or drug allergies. As an addition, the patient denied having visited any infectious disease area or contracted renal toxins or radioactive substances. The patient had no history of smoking or alcohol abuse. He denied having used illegal substances and had no history of sexually transmitted diseases. He also reported no similar illnesses in the family disease genetic history. Contrast-enhanced CT of the upper abdomen revealed the mass occupying a space in the right lobe of the liver, suggesting hepatocellular carcinoma with invasion of the portal vein and superior mesenteric vein, therefore causing embolism. Multiple lymph nodes were detected near the hepatic hilum, hepatogastric space, and abdominal aorta. Local nodular thickening of the peritoneum and omentum was also found, which, in turn, suggested spread of metastasis. Further examination revealed liver cirrhosis, splenomegaly, ascites, and varicose veins in the lower esophagus around the gastric fundus and gallbladder. On June 8, 2023, the patient was admitted to inpatient care with follow-up administration of oral donafenib tosylate as first-line cancer therapy and eltrombopag as platelet therapy. After 1 month, the anticancer effect was reported as standard deviation (SD) by imaging; however, the alpha fetoprotein (AFP) plasma level was increased. Starting July 11, 2023, the patient received therapy with hypodermic injection of envafolimab 300 mg once every 2 weeks (q2w). The patient was given 150 mg envafolimab subcutaneously twice, i.e., both arms at the lower edge of the deltoid muscle of the upper arm. At 3–4 days after the first injection, red plaques with a diameter of approximately 6 cm appeared in the injection site on both sides of the upper arm, with mild itching that gradually disappeared without treatment. On July 25, 2023, during the second cycle of immunotherapy, subcutaneous injection of envafolimab 300 mg was performed on both sides of the upper arm. The patient reported slight pain in the upper arm and in the whole body after injection. After 3 days, the pain in both sides of the upper arm aggravated. Upon examination, local bleeding and a necrotic area of approximately 10 cm of skin around the injection area on both sides were discovered (**Figures 1**, **2**). The left arm was affected more seriously than the right arm, with accompanying multiple unruptured black blisters, but the local skin temperature was not elevated. On August 4, 2023, the patient returned to continue receiving treatment. In the previous 5 days, the patient occasionally experienced unconsciousness, sleepiness, and malnutrition. Urination and defecation were normal.

**Figure 3:**
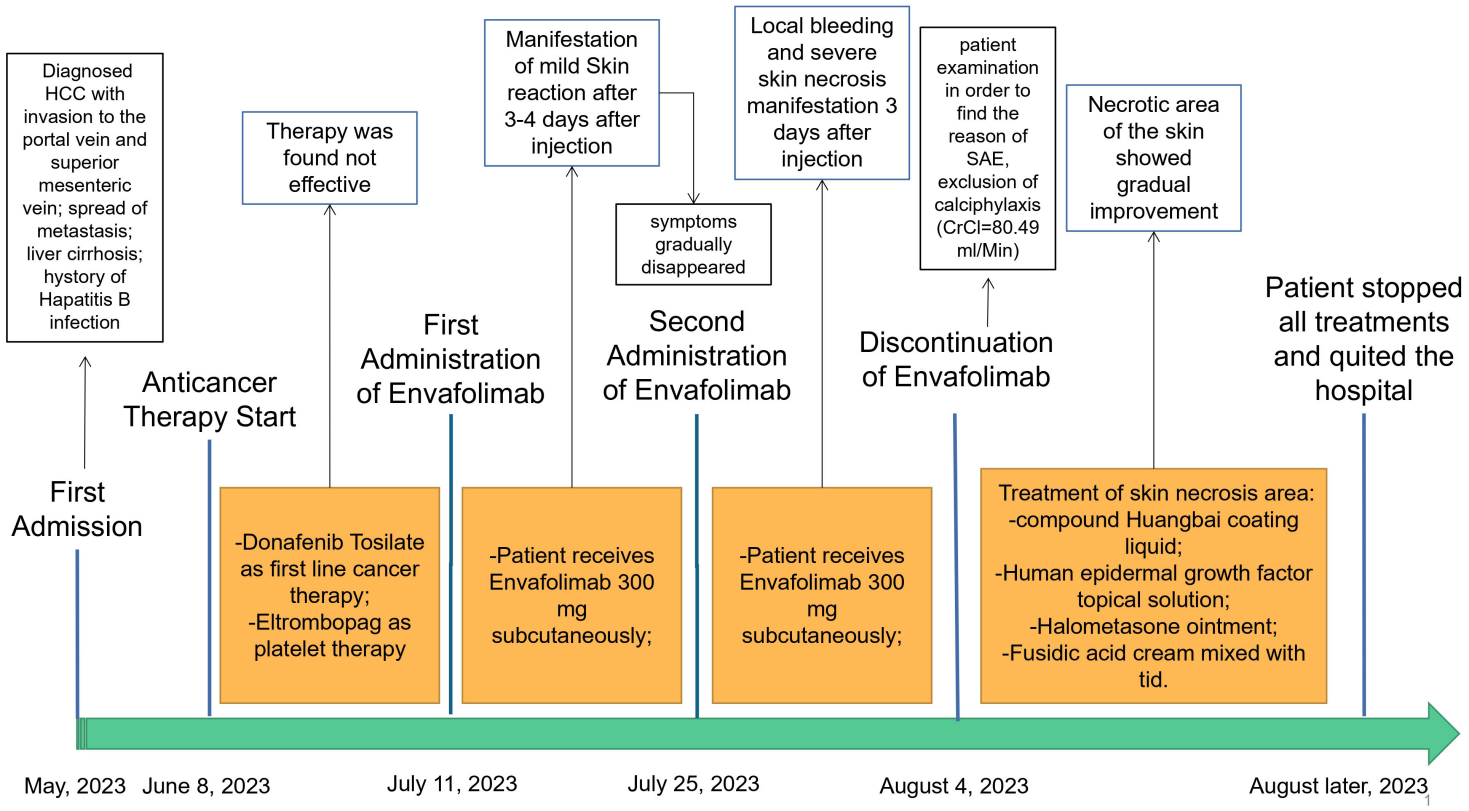
Treatment history of this case.

The same day, on physical examination, the patient’s body temperature was 36.51°C, pulse was 77 bpm, blood pressure (BP) was 103/65 mmHg, the white blood cell (WBC) count was 13.36 × 10^9^/L (range, 4–10 × 10^9^/L), neutrophil count (NEUT%) was 89.70% (range, 50–70%), and the platelet (PLT) count was 47 × 10^9^/L (range, 100–300 × 10^9^/L). Routine urine analysis revealed no abnormalities. The levels of typical markers of infection, i.e., interleukin-6 and procalcitonin, were 192.1 pg/ml (range, 0–7 pg/ml) and 1.98 ng/ml (range, 0–0.5 ng/ml), respectively. These data suggest that infection occurred. We hypothesized that it might be related to the hemorrhagic skin necrosis in both arms. The results of the common biochemistry blood tests were as follows: total bilirubin, 72.80 μmol/L (range, 3.4–20.5 μmol/L); direct bilirubin, 60.70 μmol/L (range, 0–6.8 μmol/L); albumin, 25.40 g/L (range, 35–52 g/L); glutamic oxaloacetic transaminase, 321.6 U/L (range, 0–46 U/L); alanine aminotransferase, 286.8 U/L (range, 0–33 U/L); cholinesterase, 1,298 U/L (range, 532–12,920 U/L); and plasma ammonia, 238.70 μg/L (range, 16–60 μmol/L). Five different blood coagulation tests were performed, with the following results: prothrombin time, 26.40 s (range, 104.5 s); international normalized ratio (INR), 2.40 (range, 0.8–2); activated partial prothrombin time, 55.60 s (range, 24–40 s); D-dimer, 3.14 mg/L (range, 0–0.5 mg/L); and fibrin degradation product, 18.60 μg/ml (range, 0–5 μg/L). Laboratory diagnostics of the hepatocellular carcinoma markers showed ferritin, 1,451 ng/ml (range, 30–400 ng/ml); carbohydrate antigen, 148 U/ml (range, 0–39 U/ml), and AFP >1,210 ng/ml (range, 0–7 ng/ml). The electrolyte panel test results were as follows: potassium, 3.97 mmol/L (range, 3.50–5.10 mmol/L); sodium, 130.8 mmol/L (range, 136–145 mmol/L); chloride, 100.4 mmol/L (range, 98–107 mmol/L); total calcium, 2.02 mmol/L (range, 2.15–2.50 mmol/L); and phosphorus, 0.54 mmol/L (range, 0.81–11.45 mmol/L). For renal function examination, the results were: urea, 17.10 mmol/L (range, 1.7–8.3 mmol/L); creatinine, 78 μmol/L (range, 62–106 μmol/L); uric acid, 459 μmol/L (range, 202–416 μmol/L); serum β2 microglobulin assay, 7.29 mg/L (range, 1–3 mg/L); serum cystatin, 1.54μmg/L (range, 0.63–1.25 mg/L); and creatinine clearance rate, 80.49 ml/min. Based on these results, calciphylaxis was excluded, which has been known to have a similar clinical course to the described skin necrosis in our case. Due to the absence of secretion at the site of skin necrosis, a microorganism identification culture test was not performed. After inpatient admission, the patient received magnesium isoglycyrrhetinic acid 200 mg and polyene phosphatidylcholine injection 930 mg [intravenous drip (ivgtt), qd] in combination with hepatoprotectors. Enema with lactulose oral solution 150 ml and ornithine aspartate 10 g (ivgtt, qd) were administered to decrease the plasma ammonia level. Cefuroxime axetil tablets 250 mg (second-generation cephalosporin) was chosen as the anti-infective therapy agent for this case, while 100 mg of methylprednisolone sodium succinate solution mixed with 100 ml of 0.9% sodium chloride solution for injection was used as immunosuppressive therapy. After consultation with the burn wound management department, the decision regarding treatment of the skin necrosis area included the administration of compound Huangbai coating liquid, human epidermal growth factor topical solution, halometasone ointment, and topical application of fusidic acid cream (tid). After 8 days of designated treatment, the necrotic area of the skin became scabbed, molted, and dry, showing gradual improvement. The entire treatment history can be seen in **Figure 3**. In the end, the patient’s family members decided to stop all treatments, with no consideration of alternative options for the treatment of hepatocellular carcinoma, and to quit hospitalization due to the terminal stage of the hepatocellular carcinoma and financial burden.

## Discussion

In this case, local bleeding and skin necrosis around the injection site with further symptom aggravation obviously correlated with the time of envafolimab administration. Excluding other therapeutics received by the patient at that time, we failed to explain the origin of the symptoms described in the case presentation above. Progression of the previously diagnosed hepatocellular carcinoma was also not a reason for the appearance of such symptoms. According to the Naranjo scale (5) “probable” and the five principles of causal analysis of adverse drug reactions (ADRs) in China (6), the current ADR and the use of envafolimab had a positive correlation. According to the “The Common Terminology Criteria for Adverse Events (CTCAE) version 5.0,” (7) the current ADR was classified as level 4, or serious adverse drug reaction (sADR), which requires emergency hospitalization and termination of the related drug administered.

ICPIs are monoclonal antibodies that can affect various organs and tissues due to their unique mechanism of action (8). Approximately 40% of patients experience ICPI-derived dermatologic adverse events (AEs) (9). Among these, symptoms such as papules, pruritus, and lichen-like rash are mainly classified as mild-to-moderate reactions and generally do not lead to the cessation of immunotherapy. Previously observed rare skin-related events, including dermatomyositis, interstitial granulomatous dermatitis, pityriasis rubra, and lupus, could also be considered as life-threatening (10). Approximately 5% of patients reported serious cutaneous AEs (11, 12), including Stevens–Johnson syndrome, toxic epidermal necrolysis, acute generalized exanthematous pustulosis (AGEP), and drug-induced hypersensitivity syndrome (DIHS), or drug reaction with eosinophilia and systemic symptoms (DRESS) collectively characterized by a low incidence rate, but with higher mortality. Cutaneous toxicities appear to be one of the most prevalent immune-related adverse events (irAEs) with anti-programmed cell death protein 1 (PD-1) agents (13). Manifestation of irAEs in the skin may be widely distributed in multiple areas of the body (14). However, there have been no reports of AEs at the injection site after the administration of ICPIs. Database search in CNKI, Wanfang, VIP, and PubMed on October 8, 2023, found more than 10 Chinese local and international clinical trials, including post-marketing studies on envafolimab. There were a few studies conducted on the safety and post-marketing data, which were mostly presented in case reports. Liu et al. (15) reported no sADRs during the treatment of small cell lung cancer with envafolimab in combination with other drugs, except for mild gastrointestinal adverse reactions. Yan et al. (16) mentioned one case of suspected *Pseudomonas aeruginosa*-associated pneumonia in a single patient with lung squamous cell carcinoma treated with envafolimab, but considered it to be related to a drug immune response activating effect. Li et al. reported the results of a phase II clinical trial of envafolimab (17, 18), which presented information on nine patients who experienced injection site reactions among a total of 103 patients with cancer in the study (incidence rate = 8.7%). These adverse reactions were classified as mild or moderate. Further search revealed no additional reports indicating that envafolimab caused skin toxicity, infusion reactions, and injection site reactions, among others. In the literature, the onset of skin allergic reactions, rashes, and gastrointestinal reactions have been reported to occur during the subcutaneous administration of the humanized monoclonal antibody daratumumab; however, injection site reactions were not observed (19). According to previous reports, the relationship between immunosuppressive drugs and skin-related AEs may be explained by the activation of the immune system and inflammatory response. Downregulation of the PD-1/PD-L1 pathway could cause T lymphocytes to release cytotoxic factors such as perforin and granzyme, which results in damage to the skin of affected cells (20). However, in our case, the skin necrosis was still outside the scope of our understanding of the immune mechanisms of ICI-induced cutaneous AEs. At present, no other ICPIs for subcutaneous administration are available in the market. The underlying mechanism of subcutaneous bleeding at the injection site after subcutaneous infusion requires further investigation.

Abstracting away from the toxic effects of envafolimab, a possible connection between the severe local skin necrosis in this case and the combined use of donafenib tosylate and envafolimab cannot be excluded. Donafenib tosylate is an oral small-molecule multi-kinase inhibitor that targets Raf kinase and various receptor tyrosine kinases. It also suppresses tumor cell proliferation and angiogenesis by inhibiting the vascular endothelial growth factor receptors and platelet-derived growth factor receptors, as well as Raf kinases. There is evidence that it can cause thrombocytopenia and that it increases the bleeding tendency (21). After rechecking the patient’s PLT count (47 × 10^9^/L, range = 100–300 × 10^9^/L) upon admission, we speculated that the combination of envafolimab and donafenib may have caused the injection site reaction. For oncology patients with grade 4 thrombocytopenia and, as a consequence, certain physiological coagulation dysfunction, prolonging the targeted pressure time up to 5 min to achieve hemostasis is recommended in order to decrease the incidence rate and to reduce the area of subcutaneous bleeding after injection (22).

In this paper, we present the first reported case of envafolimab-induced local skin necrosis to provide a reference for the safe clinical use of humanized monoclonal antibodies. Evidence from this report suggests standardizing the administration route and dosage of envafolimab and other ICPIs in clinical practice. We also provide options for further treatment of such complications according to our experience with this case.

## Data availability statement

The raw data supporting the conclusions of this article will be made available by the authors, without undue reservation.

## Ethics statement

The studies involving humans were approved by Ethics Committee of Yu Lin City First Hospital, IRB no. IEC-A-V1.0. The studies were conducted in accordance with the local legislation and institutional requirements. The participants provided their written informed consent to participate in this study. Written informed consent was obtained from the individual(s) for the publication of any potentially identifiable images or data included in this article.

## Author contributions

JL: Writing – original draft, Methodology, Investigation, Conceptualization. XX: Writing – original draft. TW: Writing – review & editing. WZ: Writing – review & editing. JC: Writing – review & editing, Methodology, Conceptualization. HH: Writing – review & editing, Conceptualization. MS: Writing – review & editing, Conceptualization.
